# Physical Activity Level Influences *MTHFR* Gene Methylation Profile in Diabetic Patients

**DOI:** 10.3389/fphys.2020.618672

**Published:** 2021-01-12

**Authors:** Tainá Gomes Diniz, Alexandre Sérgio Silva, Mayara Karla dos Santos Nunes, Mateus Duarte Ribeiro, João Modesto Filho, Rayner Anderson Ferreira do Nascimento, Cecília Neta Alves Pegado Gomes, Isabella Wanderley de Queiroga Evangelista, Naila Francis Paulo de Oliveira, Darlene Camati Persuhn

**Affiliations:** ^1^Post-Graduate Program in Nutrition Science, Federal University of Paraiba, Joao Pessoa, Brazil; ^2^Department of Physical Education, Federal University of Paraiba (UFPB), Joao Pessoa, Brazil; ^3^Post-Graduation Program in Development and Technological Innovation of Medicines (DITM), Federal University of Paraiba, Joao Pessoa, Brazil; ^4^Post-Graduation Program in Physical Education, Federal University of Paraiba, Joao Pessoa, Brazil; ^5^Department of Internal Medicine, Federal University of Paraiba, Joao Pessoa, Brazil; ^6^Uninassau Faculty, Mauricio de Nassau University, João Pessoa, Brazil; ^7^Nephrology Clinic, Lauro Wanderley University Hospital, Federal University of Paraiba, Joao Pessoa, Brazil; ^8^Ophthalmology Reference Center, Lauro Wanderley University Hospital, Federal University of Paraiba, Joao Pessoa, Brazil; ^9^Department of Molecular Biology, Federal University of Paraiba, Joao Pessoa, Brazil; ^10^Department of Molecular Biology and Post-Graduation Program in Nutrition Science, Federal University of Paraiba, Joao Pessoa, Brazil

**Keywords:** diabetes, *MTHFR*, methylation, physical activity (exercise), diabetes complication

## Abstract

**Introduction:**

*MTHFR* methylation status is associated with microvascular complications in diabetes, but the factors influencing this profile remain unknown.

**Objective:**

The aim of this study was to evaluate the influence of physical activity level and nutritional status on the methylation profile of the *MTHFR* gene in patients with type 2 diabetes mellitus (T2DM).

**Methods:**

A total of 111 patients, 43 men and 68 women diagnosed with DM (7.0 ± 2.3 years), answered the International Physical Activity Questionnaire (IPAQ) and underwent blood collection for biochemical analysis, DNA extraction, and *MTHFR* gene methylation profile determination.

**Result:**

The comparison of the methylation pattern showed that the partially methylated profile predominates in the insufficiently active group (85%), which does not occur in the sufficiently active group (54%) (*p* = 0.012). No differences were found in the nutritional status comparison. Logistic regression including overweight, waist circumference, gender, age, time of DM, hypertension, dyslipidemia, smoking, alcoholism, and family DM revealed that the association of the level of physical activity with methylation profile proved to be independent of these confounding variables. Considering the partially methylated profile as a result, being physically inactive favors the partially methylated *MTHFR* pattern in patients with DM.

**Conclusion:**

We concluded that insufficient physical activity is associated with partially methylated pattern of *MTHFR* promoter.

## Introduction

Research in the area of physical activity epidemiology in recent decades has revealed that there is a clear negative association between physical activity level and the emergence of various chronic diseases including diabetes mellitus (DM) (Lackland and Voeks, 2014; Aune et al., 2015; Zhao et al., 2019). These associations need to be studied in clinical, physiological, and molecular aspects. As an example, the level of physical activity (LPA) has been reported as an epigenetic modulator, affecting the methylation pattern of specific genes and global methylation, generally favoring hypomethylation (Voisin et al., 2015). However, data of this nature are still very scarce.

The *MTHFR* gene encodes an enzyme responsible for converting 5,10-methylenetetrahydrofolate to 5-methyltetrahydrofolate (MTHF), donating in this way methyl groups that are used in the synthesis of *S*-adenosyl methionine, which is the substrate for methylation reactions, including DNA. *MTHFR* activity may be influenced by single nucleotide polymorphisms that are related to the risk of individuals developing diabetes and its major chronic complications (Fekih-Mrissa et al., 2017; Chen et al., 2018; Ramanathan et al., 2019). There are evidences that *MTHFR* gene expression is influenced by its promoter’s methylation (Wang et al., 2007). Thus, detection of the methylation pattern of this gene may indicate metabolic aspects of patients. In fact, *MTHFR* methylation profile has been associated with microvascular diabetes complications diabetic retinopathy (DR) and nephropathy (DN) (Ghattas et al., 2014; Yang et al., 2016; Nunes et al., 2017).

Knowing that the environment regulates gene expression, and that people with higher levels of physical activity are better protected against both diabetes and its complications (Sailani et al., 2019), in this study, we hypothesized that the LPA of diabetics is involved in modulating the methylation profile of the *MTHFR* gene. Thus, the objective of this study was to evaluate whether the LPA and nutritional status influence the methylation profile of the *MTHFR* gene in patients with type 2 diabetes mellitus (T2DM).

## Methodology

### Participants

The study consisted of 111 individuals found in the Reference Services for the diabetic patient of the Lauro Wanderley University Hospital of the Federal University of Paraiba (HULW/UFPB) from December 2013 to November 2016. Patients with T2DM of both sexes aged more than 40 years old with at least 5 and at most 10 years of disease evolution with (*n* = 43) or without diabetic (*n* = 68) complications were included. Considering the universe of diabetics of João Pessoa (Paraíba State, Brazil), this sample size was representative of this population for a confidence level of 95% and a margin of error of 9.3%.

#### Clinical Characterization

The diagnosis of DR was based on ophthalmoscopy after pupil dilation with tropicamide 0.5%. Images of the retina (macula and central disk) were captured at an angle of 45° by a background camera. The photographs were analyzed according to the ACCORD (Action to Control Cardiovascular Risk in Diabetes) standards and recommendations (Chew et al., 2007) and the Early Treatment Diabetic Retinopathy Study (ETDRS) (Gangnon et al., 2008). DN was found by determining urinary albumin excretion in 24 h and by the patient’s clinical condition through the nephrologist physician’s anamnesis. Samples with values below 30 mg/L in 24 h of microalbuminuria were considered normal and absence of DN; samples with values equal to or above 30 mg/L in 24 h were considered indicative of DN (American Diabetes Association, 2018).

This research was approved by the Ethical Committee of the Lauro Wanderley University Hospital (João Pessoa, Paraíba State, Brazil). All ethical procedures followed the National Health Council Resolution 466/2012.

### Sociodemographic Profile

Gender, age, risk factors (smoking and drinking), and disease history (time of diagnosis of DM, family history of DM, and complications of DM) were collected.

### Anthropometric Profile

To measure weight, an electronic scale with capacity up to 150 kg and sensitivity of 100 g (Filizola^®^) was used. Subjects were weighed in lightweight, barefoot, upright posture, feet parallel and fully supported on the scale platform and with arms across their body (Ministério da Saúde, 2018). Height was measured using a stadiometer attached to the scale, which consists of a steel tube with anodized aluminum ruler, measuring from 97 to 192 cm with 0.5 cm divisions. The subjects were in an upright posture, feet together and heels against the wall. The apex of the ear and the outer corner of the eye were in a line parallel to the floor, forming a 90° angle with the stadiometer bar; thus, the horizontal bar of the stadiometer was lowered and resting on the head, allowing reading in centimeters (Ministério da Saúde, 2018). To measure abdominal circumference (AC), patients should have their legs slightly apart and the inelastic tape measure pass the umbilical scar line for the diagnosis of the result. The reference value for men is 102 cm and for Caucasian women 88 cm (National Cholesterol Education Program (NCEP) Expert Panel on Detection, Evaluation and Treatment of High Blood Cholesterol in Adults, 2002).

Body mass index (BMI) was calculated by dividing weight (in kg) by height (in meters squared). The values obtained will be categorized into low weight, normal weight, overweight, or obesity for adult individuals and thinness, normal weight, and overweight for elderly individuals according to the World Health Organization cutoff points (WHO, 2018).

### Physical Activity Level

To quantify the LPA of patients, the long version International Physical Activity Questionnaire (IPAQ) was used (Pardini et al., 2001), allowing to generate an estimate of the weekly time patients spent in physical activity at different intensities (vigorous or moderate). In different daily activities, such as work, transportation, domestic tasks, and leisure, as well as the estimated time spent in activities in the sitting position, the IPAQ was applied individually by a properly trained researcher.

According to WHO (2018) to be classified as active, at least 150 min of mild or moderate physical activity per week or at least 75 min of intense physical activity per week is required.

The results divided the patients into two groups, the active and the insufficiently active according to the IPAQ nomenclature.

### Biochemical Determinations

For the biochemical determinations, blood was collected after 12 h fasting. Enzymatic commercial tests were employed in glycemia, total cholesterol, high-density lipoprotein (HDL), and triglyceride determinations. Glycated hemoglobin was determined by immunoturbidimetry technique. All tests were performed in an automated analyzer (LabMax 240; Labtest, Lagoa Santa, Brazil) using a standardized kit and following the manufacturer’s recommended guidelines (Labtest, Lagoa Santa, Brazil).

Low-density lipoprotein (LDL) concentration was determined by Friedewald formula, where: (LDL) = (total cholesterol) − (HDL) − (triglycerides ÷ 5) (Ohkawa et al., 1979).

The cutoffs applied were: total cholesterol < 200 mg/dl, LDL < 100 mg/dl, HDL > 60 mg/dl, triglycerides < 150 mg/dl (Brazilian Society of Cardiology, 2007), blood glucose < 126 mg/dl, and glycated hemoglobin 7% (American Diabetes Association (ADA), 2010).

Dyslipidemia was defined as increased levels of total cholesterol, LDL, or triglycerides or decreased levels of HDL (Brazilian Society of Cardiology, 2007).

### Methylation Profile Analysis

Venous blood (4 ml) was collected by venipuncture into sterile tubes containing 7.2 mg K3 EDTA and stored in a freezer at −20°C for 15 days until extraction.

Blood samples were diluted in a lysis solution (10 mM Tris–HCl pH 8.5, 5 mM EDTA, 0.3 M sucrose, and 1% Triton-X-100). After centrifugation was followed at 3,200 rpm, the supernatant was discharged. This process was repeated three times. The precipitate was then resuspended in a second lysis solution [10 mM Tris–HCl pH 8, 0.5% sodium dodecyl sulfate (SDS), 5 mM EDTA] and 0.2 μg proteinase K (Invitrogen, Carlsbad, CA, United States) and incubated at 55°C in a water bath. After 7 h of incubation 500 μl of a 1 mM EDTA 7.5 M ammonium acetate solution was added followed by vigorous mixing. The mixture was centrifuged for 10 min at 14,000 × *g* at 4°C, and 700 μl of the supernatant was transferred to a new tube where DNA precipitation with 540 μl of isopropanol was performed. The DNA precipitate was washed with 70% ethanol, centrifuged (12,000 × *g* for 5 min), dried, and resuspended in Tris–EDTA pH 8.0 buffer (Miller et al., 1988).

The extracted DNA was converted (500 ng) by sodium bisulfite using the EZ DNA Methylation^TM^ Kit (Zymo Research) according to the manufacturer’s instructions (conversion efficiency ≥ 99%). The principle of the technique is to transform unmethylated cytosine into uracil without causing alteration in methylated cytosine from the DNA sample. Different methylation patterns will be recognized through PCRs that employ specific pairs of primers for each methylation condition (methylated and unmethylated). Methylated and unmethylated DNA (Cells-to-CpG^TM^ Methylated and Unmethylated DNA Control Kit; Life Technologies) were used as controls, which were modified, as previously mentioned, and amplified by PCR as control of the reactions with primers for the methylated and unmethylated conditions, respectively.

For each methylation-specific PCR, a mixture containing 100 ng bisulfite-transformed DNA (both controls and samples), 0.7 μl (7 μM) from each methylated (sense: 5′-tagatttaggtacgtgaagtagggtagac-3′ and antisense: 5′-gaaaaactaataaaaaaaccgaca-3′) and unmethylated target-specific primer (sense: 5′-tttaggtatgtgaagtagggtagatgt-3′ and antisense: 5′-caaaaaactaataaaaaaccaacaaa-3′) as previously described (Khazamipour et al., 2009) and 1 × Go Taq Hot Start Green Master Mix (Promega Corporations, Madison, WI, United States) in a 25 μl final reaction was performed. The annealing temperature was 58°C for 40 s and 40 cycles. Amplified PCR samples were loaded (7 μl) on 3% red gel agarose gels and electrophoresed. The DNA bands were visualized in ultraviolet light transluminator.

### Statistical Analysis

Frequency distribution was used for categorical variables, as well as descriptive statistics (mean, standard deviation) for continuous variables. Chi-square test was used to test the relationship between methylation profile and physical activity level, along with a binary logistic regression. Statistical analyses were performed using SPSS version 24.0 (SPSS, Inc., Chicago, IL, United States) and *p* < 0.05 as statistical significance.

## Results

### Population Characteristics

Participants were 43 males and 68 females, mean age 58.2 ± 9.4 years with a diagnosis of DM at about 7.0 ± 2.3 years, with or without microvascular complications. The epidemiological, clinical, anthropometric, and metabolic characteristics of the studied groups are shown in Table 1. It was observed that most individuals were hypertensive, dyslipidemic, and overweight or obese, and this occurred in both groups (active and insufficiently active). The group classified as active had a lower percentage of males (*p* = 0.01), dyslipidemics (*p* = 0.01), smokers (*p* = 0.02), and hypertension carriers (0.01). Active and insufficiently active groups were similar in the distribution of elderly people percentual, complications (DR and DN), overweight/obesity, DM duration, and treatment in course.

**TABLE 1:** Characteristics of diabetic patients as a function of levels of physical activity.

	Active *n* = 91		Insufficiently active *n* = 20		
		
	*n*	%	*N*	%	*P*
<60 years	50	54.9	8	40	0.317
Male	32	35.2	11	55	0.01*
Dyslipidemia	69	75.8	18	90	0.01*
Smoking habit	7	7.7	2	10	0.02*
Alcoholism	13	14.3	1	5	0.01*
Overweight/obesity	77	84.6	15	75	0.070
Hypertension	62	68.1	17	85	0.01*
DM duration	87	95.6	20	100	0.09
Insulin use	21	23.1	1	5	0.187
Diabetes in the family	61	67	14	70	0.022*
DM complications	43	47.2	10	50	0.09
Diabetic retinopathy	20	22	5	25	0.07
Diabetic nephropathy	30	33	8	40	0.26

*DM diagnosis time ≥ 5 years. Test *x*^2^, *p* < 0.05. João Pessoa, 2016 (*n* = 111).

In the present study, patients were grouped according to the IPAQ: active (*n* = 91) and the insufficiently active (*n* = 20) with a mean time of physical activity of 949.3 ± 850.7 and 33.2 ± 51.7 min, respectively.

**FIGURE 1:**
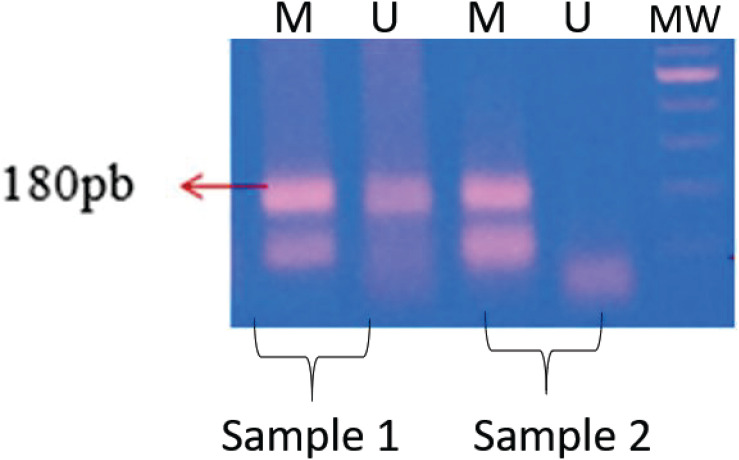
Methylation profile of the *MTHFR* gene. Methylation patterns of *MTHFR* gene samples. Sample 1, partially methylated pattern; sample 2, fully methylated pattern; MW, 100 bp pattern. M = methylated/U = unmethylated.

The methylation profile was identified by agarose gel electrophoresis as shown in Figure 1. The presence of the band with only the pair of primers for the methylated condition results in a methylated profile. The presence of bands resulting from the amplification reactions with the two pairs of primers (methylated and non-methylated) simultaneously is related to the partially methylated profile. We did not observe the amplification of any sample only with the unmethylated condition, so we do not have this category of methylation profile for this gene in the population studied.

As the primer pairs are specific to each tested condition (methylated and non-methylated), only samples after transformation by the bisulfite technique were tested. Commercial methylated and non-methylated controls were used to make sure that the efficiency of the transformation process and the amplification reaction worked properly.

**FIGURE 2:**
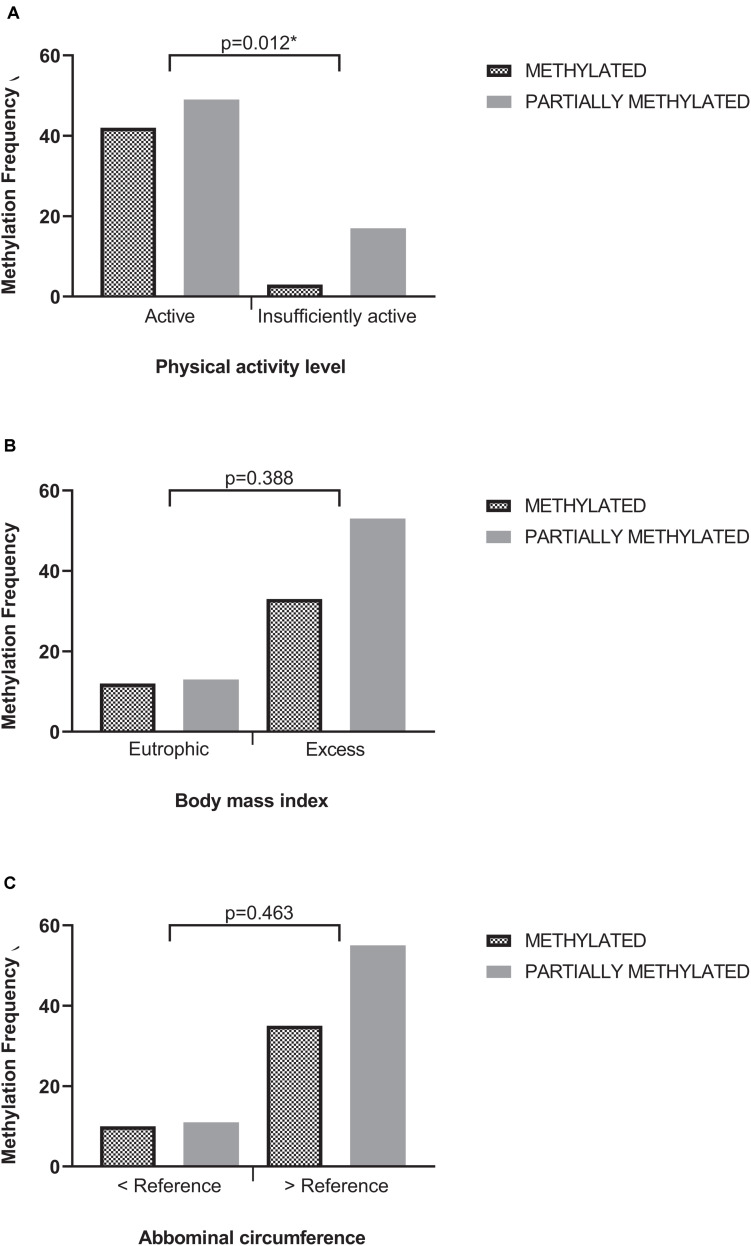
Methylation profile according to physical activity level **(A)**, body mass index **(B)**, and abdominal circumference **(C)**. Chi-square test; *p* = value of significance. AC, Abdominal circumference.

Among the samples, 66 (59.5%) presented the methylated profile, and 45 (45.5%) presented the partially methylated profile. Samples with the unmethylated profile of the *MTHFR* gene were not present (Figure 2A).

Comparing the methylation profile in relation to the LPA, it was possible to observe a balance between the methylated and partially methylated states among those classified as active (respectively, 46 and 54%). On the other hand, among the insufficiently active, there was an imbalance in distribution, with 85% of this group with partially methylated profile to only 15% with methylated profile. The chi-square test showed that this difference in distribution was statistically significant (Figure 2A). According to odds ratio (OR), DM patients with inactive profile have a partially methylated *MTHFR* gene pattern.

Figures 2B,C shows that the methylation profile of the MTHFR was not influenced by the anthropometric parameters BMI (*p* = 0.388) and waist circumference (*p* = 0.463) once methylated and partially methylated samples were equally distributed in the BMI and AC categories in the population studied.

Table 2 presents the result of logistic regression, in which the methylation profile was considered the dependent variable and as independent variables gender, age, time of DM, hypertension, dyslipidemia, smoking, alcoholism, family DM, LPA, presence of microvascular complications (DR and/or DN), overweight, and AC. This test revealed that none of the independent variables besides LPA was found to influence the methylation profile, suggesting that LPA is an independent factor influencing the methylation profile. The OR test revealed that being physically active represents a protection factor against the partially methylated profile outcome in patients with DM.

**TABLE 2:** Logistic regression model verifying the influence of LPA and confounding variables on the occurrence of *MTHFR* gene methylation profile.

	OR	Confidence interval	*p*-value
Age	0.874	0.330–2.310	0.785
Male	0.767	0.302–1.946	0.576
Hypertension	0.668	0.254–1.754	0.413
Hb1AC	0.652	0.206–2.061	0.466
Blood glucose	1.413	0.496–4.023	0.517
Total cholesterol	1.081	0.328–3.564	0.898
HDL	0.939	0.196–4.489	0.937
LDL	1.352	0.445–4.110	0.595
Triglycerides	1.436	0.500–4.130	0.502
LPA	6.185	1.476–25.911	0.013*
Sex	0.352	0.300–1.906	0.553
Smoking	1.661	0.407–6.776	0.479
Alcoholism	0.365	0.075–1.897	0.227
Diabetes in the family	1.688	0.664–4.291	0.271
DM time	0.918	0.353–2.383	0.860
Insulin use	0.604	0.224–1.630	0.319
Overweight	0.643	0.198–2.083	0.462
AC	1.292	0.338–4.560	0.707

LPA, Physical activity level; AC, Abdominal circumference. *p < 0.05.

No results were significant when comparing the methylation profile with the lipid and glycemic profile of the patients.

## Discussion

According to this study, the lack of physical activity in diabetics with a diagnosis time of 5–10 years is associated with the *MTHFR* promoter partially methylated profile, and this association was independent of the nutritional status.

It is well established in the literature that exercise is an important protective factor for diabetes (Esteghamati et al., 2014; Li et al., 2015; Roya et al., 2019). There is a direct beneficial effect of exercise on β cell function (Li et al., 2015). It is also consensus that increased homocysteine (Hcy) is an independent risk factor for cardiovascular disease and is modifiable by nutrition and exercise (Bostom et al., 1999; Ganguly and Alam, 2015).

Deminice et al. (2016) provided evidence that acute exercise increases plasma Hcy concentration regardless of the duration or intensity of exercise performed. An analysis of physical intervention in groups of pre-diabetic and normoglycemic individuals also found a relationship between high intensity physical activity and increased Hcy levels and impact on other transulfurization pathway metabolites impacting elevated glutathione levels possibly due to a metabolic response to increased production of reactive oxygen species under conditions of intense physical activity (Lee et al., 2019). Similar results were found in experimental studies using acute swimming exercise in rats that showed acute exercise increased the flow of transmethylation reactions that elevate Hcy formation (Riberio et al., 2018). Evidence of the relationship between the effects of physical activity and the metabolism of methyl groups was also demonstrated in the analysis of gene polymorphisms related to the transfer of methyl groups in elite athletes (Terruzzi et al., 2011). A predominance of genotypes that form less functional versions of enzymes involved in DNA methylation, including MTHFR, has been demonstrated in elite athletes (Terruzzi et al., 2011). Similar results were found in strength and speed athletes (Zarebska et al., 2014). Thus, studies that analyzed polymorphism found a relationship between less functional versions of *MTHFR* and differential physical performance.

In this work, we evaluated the methylation profile of the *MTHFR* gene, which plays a central role in the methionine Hcy remethylation pathway. We identified predominance of partially methylated profile, which possibly leads to higher levels of expression than the fully methylated profile, in individuals with insufficient levels of physical activity. Knowing that *MTHFR* is involved in remethylation (and consequent removal) of Hcy, we can speculate that there will be less expression of the enzyme (due to promoter hypermethylation) and consequently higher levels of Hcy in the most active individuals. Assuming that (1) there are evidences that methylation impacts *MTHFR* gene expression (Wang et al., 2007), (2) in general, DNA promoter methylation is a silencing expression mechanism, (3) the methylated profile predominated in the physically active, (4) there are relations described between high physical activity level and Hcy, and (5) the involvement of *MTHFR* on the Hcy metabolism, it is possible to suggest that methylation is a mechanism of *MTHFR* expression control mediated by physical activity.

In the context of diabetes complications, while the hypermethylated *MTHFR* profile has been associated with DR (Nunes et al., 2017), in the DN, the results are contradictory, because while hypermethylation predominated in patients with end-stage renal disease (Ghattas et al., 2014), diabetics with initial DN or in less advanced stages had a higher occurrence of the non-methylated profile (Yang et al., 2016; Nunes et al., 2017). Hypermethylation of the *MTHFR* promoter in blood samples has been linked to other relevant clinical conditions, such as women who have had recurrent miscarriages (Mishra et al., 2019) and mothers of Down syndrome patients (Coppedè et al., 2016). In both cases, the method of analyzing methylation employed quantitative techniques, making it difficult to compare with the qualitative data presented here.

It is important to understand the factors that modify the *MTHFR* methylation pattern, especially those that can be modified (e.g., physical activity), taking into account that DNA methylation is a potentially reversible epigenetic mechanism. Therefore, it is possible from these relationships to identify strategies for preventing or monitoring chronic complications in diabetes and other clinical conditions related to specific profiles.

Some aspects of this work need to be considered. First, the group of patients we studied is made up of type 2 diabetics, and comparisons are made with studies that analyzed athletes. A second aspect is that, in this work, the LPA was accessed through a structured questionnaire, and there was no physical intervention; in addition, there are other ways to measure this variable, such as the use of accelerometers. Third, we analyzed the methylation profile and not the quantification, which would offer more accurate results.

In this study, we found a higher prevalence of hypertensive patients in the inactive group, which corroborates with other studies (Jurik and Stastny, 2019; Sharman et al., 2019; Alpsoy, 2020) that demonstrated the association between aerobic and resistance exercises and reduced blood pressure (BP). The guidelines of the Canadian Journal of Cardiology (Rabi et al., 2020) strongly recommend aerobic exercises of moderate intensity for at least 30 min on at least 3 days of the week or resistance exercises on 2–3 days of the week, aiming at BP control.

Dyslipidemia in this study was more frequent among the inactive, but a high percentage (75.8%) was also found in the active group. The effect of physical activity on the control of lipid metabolism and consequently of circulating levels has a wide repercussion in the literature (Gordon et al., 2014; Mann et al., 2014; Fikenzer et al., 2018).

The contribution of this work is to demonstrate that there is a relationship between *MTHFR* methylation, a molecular marker previously related to microvascular complications, and activity levels in diabetic individuals. The data presented here aggregate molecular information at the epigenetic level on the well-known relationship between physical activity and diabetes prevention.

Studies of exercise physiology and biochemistry have previously demonstrated the relationship between physical activity and Hcy levels. They also demonstrated an association between physical exercise and global hypomethylation and also a relationship between polymorphisms that impact *MTHFR* activity and outstanding physical performance. Our study brings the unprecedented approach of evaluating the methylation profile of the *MTHFR* gene in the context of physical activity, showing that physical inactivity favors the occurrence of the partially methylated profile.

This is a first observation that opens the way for more detailed investigations, exploring the biological and physiological aspects of the association. The association found in the present study of descriptive and cross-sectional characteristics encourages the development of an intervention study to test the effect of a physical training program on the methylation profile of previously sedentary diabetics.

## Conclusion

We concluded that insufficient physical activity is associated with partially methylated pattern of *MTHFR* promoter in patients with diabetes. Our results encourage further studies focused on determining if this result is affected by the metabolic profile of participants with diabetes.

## Data Availability Statement

The raw data supporting the conclusions of this article will be made available by the authors, without undue reservation.

## Ethics Statement

The studies involving human participants were reviewed and approved by Comitê de Ética em Pesquisa do Hospital Universitário Lauro Wanderley. The patients/participants provided their written informed consent to participate in this study.

## Author Contributions

TD performed statistical analysis, interpretation of results, and writing of the manuscript. MR performed statistical analysis. AS analyzed data from the manuscript. MS participated in data collection, biochemical analysis, and molecular biology experiments. JF participated in data collection and performed endocrinological consultation in individuals. RN participated in data collection and biochemical analysis. CG participated in data collection and carried out individual consultations. IQ participated in data collection and performed ophthalmological consultation in individuals. NO participated in experiments in molecular biology. DP participated in all stages of the research (study design and logistics, data collection, molecular biology experiments, interpretation of results, statistical analysis, and writing of the manuscript). All authors read and approved the final manuscript.

## Conflict of Interest

The authors declare that the research was conducted in the absence of any commercial or financial relationships that could be construed as a potential conflict of interest.

## References

[B1] Alpsoy S. (2020). Exercise and hypertension. *Adv. Exp. Med. Biol.* 1228 153–167. 10.1007/978-981-15-1792-1_10 32342456

[B2] American Diabetes Association (2018). Microvascular Complications and foot care: standards of medical care in diabetesd-2018. *Diabetes Care* 41 105–118. 10.2337/dc18-S010 29222381

[B3] American Diabetes Association (ADA) (2010). Standards of medical care in diabetes-2010. *Diabetes Care* 33 11–61. 10.2337/dc10-S011 20042772 PMC2797382

[B4] Aune D. Norat T. Leitzmann M. Tonstad S. Vatten L. J. (2015). Physical activity and the risk of type 2 diabetes: a systematic review and dose-response meta-analysis. *Eur. J. Epidemiol.* 30 529–542. 10.1007/s10654-015-0056-z 26092138

[B5] Bostom A. G. Silbershatz H. Rosenberg I. H. Selhub J. D’Agostino R. B. Wolf P. A. (1999). Nonfasting plasma total homocysteine levels and all-cause and cardiovascular disease mortality in elderly Framingham men and women. *Arch. Intern. Med.* 159 1077–1080. 10.1001/archinte.159.10.1077 10335684

[B6] Brazilian Society of Cardiology (2007). IV brazilian guideline on dyslipidemias and prevention of atherosclerosis. *Arq. Bras. Cardiol.* 88 2–19. 17515982 10.1590/s0066-782x2007000700002

[B7] Chen D. Wang J. Dan Z. Shen X. Ci D. (2018). The relationship between methylenetetrahydrofolate reductase C677T polymorphism and diabetic retinopathy: A meta-analysis in multiethnic groups. *Ophthalmic Genet.* 39 200–207. 10.1080/13816810.2017.1401087 29182429

[B8] Chew E. Y. Ambrosius W. T. Howard L. T. Greven C. M. Johnson S. Danis R. P. (2007). Rationale, design, and methods of the action to control cardiovascular risk in diabetes eye study (ACCORD-EYE). *Am. J. Cardiol.* 99 103i–111i. 10.1016/j.amjcard.2007.03.028 17599420

[B9] Coppedè F. Stoccoro A. Tannorella P. Gallo R. Nicolì V. Migliore L. (2016). Increased *MTHFR* promoter methylation in mothers od down syndrome individuals. *Mutat. Res. Fundament. Mol. Mech. Mutag.* 787 1–6. 10.1016/j.mrfmmm.2016.02.008 26926955

[B10] Deminice R. Ribeiro D. F. Frajacomo F. T. T. (2016). The effects of acute exercise and exercise training on plasma homocysteine: a meta-analysis. *PLoS One* 11:e0151653. 10.1371/journal.pone.0151653 26986570 PMC4795785

[B11] Diniz T. G. (2020). *Influência do nível de atividade física e estado nutricional no perfil de metilação do gene MTHFR em pacientes diabéticos*. Dissertation/Master’s Thesis, Universidade Federal da Paraíba, João Pessoa, Paraíba, Brazil Available online at: https://repositorio.ufpb.br/jspui/handle/123456789/18344

[B12] Esteghamati A. Etemad K. Koohpayehzadeh J. Abbasi M. Meysamie A. Noshad S. (2014). Trends in the prevalence of diabetes and impaired fasting glucose in association with obesity in Iran: 2005–2011. *Res. Clin. Pract. Diab.* 103 319–327. 10.1016/j.diabres.2013.12.034 24447808

[B13] Fekih-Mrissa N. Mrad M. Ibrahim H. Akremi I. Sayeh A. Jaidane A. (2017). Methylenetetrahydrofolate reductase (*MTHFR*) (C677T and A1298C) polymorphisms and vascular complications in patients with type 2 diabetes. *Can. J. Diabetes.* 41 366–371. 10.1016/j.jcjd.2016.11.007 28341195

[B14] Fikenzer K. Fikenzer S. Laufs U. Werner C. (2018). Effects of endurance training on serum lipids. *Vas. Pharmacol.* 101 9–20. 10.1016/j.vph.2017.11.005 29203287

[B15] Gangnon R. E. Davis M. D. Hubbard L. D. Aiello L. M. Chew E. Y. Ferris F. L. III (2008). A severity scale for diabetic macular edema developed from ETDRS data. *Investigat. Ophthalmol. Vis. Sci.* 49 5041–5047. 10.1167/iovs.08-2231 18539929 PMC3028439

[B16] Ganguly P. Alam S. F. (2015). Role of homocysteine in the development of cardiovascular disease. *Nutr. J.* 14:6. 10.1186/1475-2891-14-6 25577237 PMC4326479

[B17] Ghattas M. El-Shaarawy E. Mesbah N. Abo-Elmatty D. (2014). DNA methylation status of the methylenetetrahydrofolate reductase gene promoter in peripheral blood of end-stage renal disease patients. *Mol. Biol. Rep.* 41 683–688. 10.1007/s11033-013-2906-7 24363223

[B18] Gordon B. Chen S. Durstine J. L. (2014). The effects of exercise training on the traditional lipid profile and beyond. *Curr. Sports Med. Rep.* 13 253–259. 10.1249/JSR.0000000000000073 25014391

[B19] Jurik R. Stastny P. (2019). Role of nutrition and exercise programs in reducing blood pressure: a systematic review. *J. Clin. Med.* 8:1393. 10.3390/jcm8091393 31492032 PMC6780911

[B20] Khazamipour N. Noruzinia M. Fatehmanesh P. Keyhanee M. Pujol P. (2009). *MTHFR* promoter hypermethylation in testicular biopsies of patients with non-obstructive azoospermia: the role of epigenetics in male infertility. *Hum. Reprod.* 24 2361–2364. 10.1093/humrep/dep194 19477879

[B21] Lackland D. T. Voeks J. h. (2014). Metabolic syndrome and hypertension: regular exercise as part of lifestyle management. *Curr. Hypertens Rep.* 16:492. 10.1007/s11906-014-0492-2 25190022

[B22] Lee S. Olsen T. Vinknes K. J. Refsum H. Gulseth H. L. Birkeland K. I. (2019). Plasma sulphur-containing amino acids, physical exercise and insulin sensitivity in overweight dysglycemic and normal weight normoglycemic men. *Nutrients* 11:10. 10.3390/nu11010010 30577516 PMC6356487

[B23] Li T. He S. Liu S. Kong Z. Wang J. Zhang Y. (2015). Effects of different exercise durations on Keap1-Nrf2-ARE pathway activation in mouse skeletal muscle. *Free Radic. Res.* 49 1269–1274. 10.3109/10715762.2015.1066784 26118597

[B24] Mann S. Beedie C. Jimenez A. (2014). Differential effects of aerobic exercise, resistance training and combined exercise modalities on cholesterol and the lipid profile: review, synthesis and recommendations. *Sports Med.* 44 211–221. 10.1007/s40279-013-0110-5 24174305 PMC3906547

[B25] Miller S. A. W. Dykes D. D. Polesky H. F. (1988). A simple salting out procedure for extracting DNA from human nucleated cells. *Nucleic Acids Res.* 16 1215. 10.1093/nar/16.3.1215 3344216 PMC334765

[B26] Ministério da Saúde (2018). *SISVAN – FOOD AND NUTRITIONAL SURVEILLANCE SYSTEM: Basic Guidelines for the Collection, Processing, Data Analysis and Information in Health Services. Ministry of Health, Secretariat of Health Care, Department of Primary Care. – Brasilia: Ministry of Health, 2011. 76.* Available online at: http://bvsms.saude.gov.br/bvs/publicacoes/orientacoes_coleta_analise_dados_antropometricos.pdf (acessed November 25, 2018).

[B27] Mishra J. Talwar S. Kaur L. Chandiok K. Yadav S. Puri M. (2019). Differential global and *MTHFR* gene specific methylation patterns in preeclampsia and recurrent miscarriages: a case-control study from North India. *Gene* 704 68–73. 10.1016/j.gene.2019.04.036 30986448

[B28] National Cholesterol Education Program (NCEP) Expert Panel on Detection, Evaluation and Treatment of High Blood Cholesterol in Adults (2002). Third report of cholesterol education program (NCEP) expert panel on detection, evaluation, and treatment of high blood cholesterol in adults (Adult Treatment Panel III) 2000. NIH publication n. 01-3670. *Circulation* 106 3143–3421. 10.1161/circ.106.25.3143 12485966

[B29] Nunes M. K. S. Silva A. S. Evangelista I. W. Q. Filho J. M. Gomes C. N. A. P. do Nascimento R. A. F. (2017). Hypermethylation in the promoter of the *MTHFR* gene is associated with diabetic complications and biochemical indicators. *Diabetol. Metab Syndr.* 9:84. 10.1186/s13098-017-0284-3 29075332 PMC5648437

[B30] Ohkawa H. Ohishi N. Yagi K. (1979). Assay for lipid peroxides in animal tissues by thiobarbituric acid reaction. *Anal. Biochem.* 95 351–358. 10.1016/0003-2697(79)90738-3 36810

[B31] Pardini R. Matsudo S. Araújo T. Matsudo V. Andrade E. Braggio G. (2001). Validation of the international physicalactivity questionaire (IPAQ version 6): pilot study in Brasilian Young adults. *Ver. Bras. Ciên. E Mov.* 9 45–51.

[B32] Rabi D. M. McBrien K. A. Sapir-Pichhadze R. Nakhla M. Ahmed S. B. Dumanski S. M. (2020). Hypertension Canada’s 2020 comprehensive guidelines for the prevention, diagnosis, risk assessment, and treatment of hypertension in adults and children. *Can. J. Cardiol.* 36 596–624. 10.1016/j.cjca.2020.02.086 32389335

[B33] Ramanathan G. Harichandana B. Kannan S. Elumalai R. Sfd P. (2019). Association between end-stage diabetic nephropathy and *MTHFR* (C677T and A1298C) gene polymorphisms. *Nephrology (Carlton)* 24 155–159. 10.1111/nep.13208 29227003

[B34] Riberio D. F. Cella P. S. Silva L. E. C. M. Jordao A. A. Deminice R. (2018). Acute exercise alters homocysteine plasma concentration in an intensity-dependent manner due increased methyl flux in liver of rats. *Life Sci.* 196 63–68. 10.1016/j.lfs.2018.01.003 29307522

[B35] Roya S. F. Fatemeh R. Kamran T. Behrooz H. Yahya P. Mehdi M. (2019). Prevalence, awareness, treatment, control, and the associated factors of diabetes in an iranian kurdish population. *J Diabetes Res.* 2019:5869206. 10.1155/2019/5869206 31565657 PMC6745166

[B36] Sailani R. Halling J. F. Møller D. H. Lee H. Plomgaard P. Pilegaard H. (2019). Lifelong physical activity is associated with promoter hypomethylation of genes involved in metabolism, myogenesis, contractile properties and oxidative stress resistance in aged human skeletal muscle. *Sci. Rep.* 9:3272. 10.1038/s41598-018-37895-8 30824849 PMC6397284

[B37] Sharman J. E. Smart N. A. Coombes J. S. Stowasser M. (2019). Exercise and sport science australia position stand update on exercise and hypertension. *J. Hum. Hypertens* 33 837–843. 10.1038/s41371-019-0266-z 31582784

[B38] Terruzzi I. Senesi P. Montesano A. La Torre A. Alberti G. Benedini S. (2011). Genetic polymorphisms of the enzymes involved in DNA methylation and synthesis in elite athletes. *Physiol. Genomics* 43 965–973. 10.1152/physiolgenomics.00040.2010 21673074

[B39] Voisin S. Eynon N. Yan X. Bispo D. J. (2015). Exercise training and DNA methylation in humans. *Acta Physiol. (Oxf.)* 213 39–59. 10.1111/apha.12414 25345837

[B40] Wang L. Zhang J. Wang S. (2007). Demethylation in the promoter region of *MTHFR* gene and its mRNA expression in cultured human vascular smooth muscle cells induced by homocysteine. *Wei Sheng Yan Jiu* 36 291–294. Chinese. 17712942

[B41] WHO (2018). *Obesity: Preventing and Managing the Global Epidemic, 2000.* Available online at: https://www.who.int/nutrition/publications/obesity/WHO_TRS_894/en/ (acessed June 11, 2018).

[B42] Yang X. H. Cao R. F. Yu Y. Sui M. Zhang T. Xu J. Y. (2016). A study on the correlation between *MTHFR* promoter methylation and diabetic nephropathy. *Am. J. Transl. Res.* 8 4960–4967. 27904696 PMC5126338

[B43] Zarebska A. Ahmetov I. I. Sawczyn S. Weiner A. S. Kaczmarczyk M. Ficek K. (2014). Association of the *MTHFR* 1298A>C (rs1801131) polymorphism with speed and strength sports in Russian and Polish athletes. *J. Sports Sci.* 32 375–382. 10.1080/02640414.2013.825731 24015812

[B44] Zhao M. Veeranki S. P. Li S. Steffen L. M. Xi B. (2019). Beneficial associations of low and large doses of leisure time physical activity with all-cause, cardiovascular disease and cancer mortality: a national cohort study of 88,140 US adults. *Br. J. Sports Med.* 53 1405–1411. 10.1136/bjsports-2018-099254 30890520

